# Evaluation of the weekly disease surveillance system for epidemic-prone diseases in Makonde District, Zimbabwe 2020: a descriptive cross-sectional study

**DOI:** 10.11604/pamj.2022.43.132.35001

**Published:** 2022-11-09

**Authors:** Kudzai Madamombe, Mujinga Karakadzai, Gift Masoja, Tapiwa Dhliwayo, Tsitsi Juru, Addmore Chadambuka, Emmanuel Govha, Notion Gombe, Mufuta Tshimanga

**Affiliations:** 1 Department of Primary Health Sciences, Family Medicine/Global and Public Health Unit, University of Zimbabwe, Harare, Zimbabwe,; 2 Zimbabwe Community Health Intervention Research, Harare, Zimbabwe,; 3 Zimbabwe Ministry of Health and Child Care, Mashonaland West, Zimbabwe,; 4 Africa Field Epidemiology Network, Harare, Zimbabwe

**Keywords:** Weekly disease surveillance, Makonde, Zimbabwe

## Abstract

**Introduction:**

the weekly disease surveillance system (WDSS) is a tool used to provide an early warning of potential public health threats in Zimbabwe. In 2020, Makonde District failed to detect an anthrax outbreak at one of its facilities, this untimely and poor response of the district increased morbidity. We evaluated the weekly disease surveillance system to determine reasons for missing outbreaks and determine the usefulness of the system in the detection of outbreaks.

**Methods:**

we conducted descriptive cross-sectional study using updated Centres for Disease Control and Prevention guidelines for surveillance system evaluation. We recruited 46 health workers. A structured pretested interviewer-administered questionnaire was used to collect data on reasons for missing outbreaks, knowledge, usefulness and surveillance system attributes. Data were cleaned and bivariate analysis was conducted.

**Results:**

health workers found the system simple (85%), acceptable (75%) and flexible (60%). However, we found only 5 (11%) health workers could correctly describe the surveillance system, only 2 (3%) were trained in disease surveillance, only 31 (65%) sent data on time, 57% of clinics had stock outs of forms, 60% of forms had entries with 100% of the data filled out and 22 (46%) of health workers analysed the data gathered and used it in meetings.

**Conclusion:**

the surveillance system was simple, flexible, acceptable, but unstable, untimely and not useful. There was poor knowledge on the surveillance system, health workers were not trained on disease surveillance, and quality of data was poor. Health workers should be trained in surveillance and data validation and adequate reporting tools provided.

## Introduction

The weekly disease surveillance system (WDSS) is a surveillance tool used to provide an early warning of potential public health threats and program monitoring functions that may be disease-specific or multi-disease in nature [1]. The system was adopted by Zimbabwe in 1992 to monitor several local diseases and the system has battled with inadequate and delayed reporting from facilities to the district and national levels because to an unpredictable macroeconomic climate [2]. In order to provide for the conditions for improvement of the health and quality of life and healthcare [3], the WDSS was set up to monitor nineteen diseases and events of public health importance including infectious diseases such as cholera and anthrax as well as monitoring maternal deaths. These diseases and events are often prone to rapid spread hence system serves as an early warning system for potential public health hazards by tracking disease trends on a weekly basis. The efficient running of the WDSS relies on the completeness of the reports and demonstration of usefulness as evidenced by the use of surveillance data for policy, and detection of outbreaks [4].

A review of the Makonde District weekly disease surveillance data in district health information system (DHIS2) in 2020 showed that the district failed to detect an anthrax outbreak at one of its facilities between weeks 28 and 29 which recorded an upsurge in cases from zero cases in week 27 to six cases in week 28. The district was not aware of the deviation hence neither an outbreak report nor was an investigation done. Anthrax is a highly infectious notifiable disease. The bacterium that causes it can develop spores that can survive in pasture lands for more than sixty years. The failure to identify, report and investigate the outbreak in time resulted in another upsurge of anthrax cases at the clinic in week 36 where the facility recorded 7 anthrax cases. One of the cases developed severe complications requiring surgical care. The untimely and poor response of the district increased morbidity. This study was therefore set out to evaluate the performance of the WDSS in Makonde District, to assess knowledge of the system among health workers, evaluate the system's attributes and determine the usefulness of the surveillance system in the detection and control of outbreaks in Makonde District.

**Figure 1:**
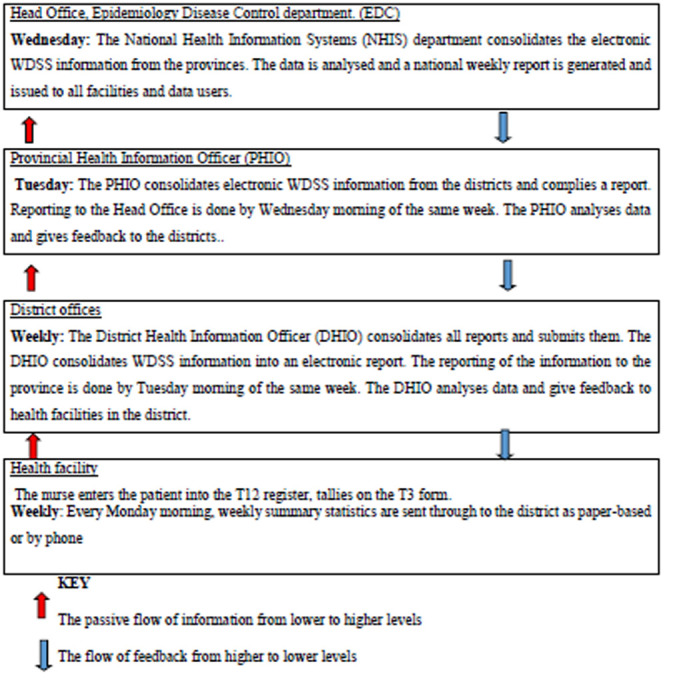
WDSS information flow chart from facility to national

**Operation of the WDSS:** reporting on the WDSS starts at the lowest primary healthcare facility in that area. The reporting week begins a minute past midnight every Monday morning and ends at midnight on Sunday. Each facility has a numbered system of forms which record the various activities done there. The series of forms filled out at each facility are denoted with letter T followed by a number. At the primary health facility, the patient is first entered into the outpatient department and have their demographic information, presenting complaints, diagnosis, and management recorded into the facility register which is also called the Tally form 12 (T12) and simultaneously the weekly summary sheet of the each of the various conditions' patients present with also called the Tally form 3 (T3) is tallied. The T3 form summaries all the conditions that were recorded at the facility that week. The summarized information is sent to the district health information officer (DHIO) by 9 o’clock on Monday morning. The DHIO then compiles and aggregates the data for all the facilities then compiles a district report. The information is sent by the DHIO to the provincial health information officer (PHIO) by Tuesday 9 o’clock. The PHIO will in turn compile and aggregate the district reports into a provincial report. By Wednesday 9 o’clock the provincial report is sent to the deputy director of health information (DHI) in the national health information office. Weekly bulletins are issued at all levels and disseminated to all relevant health workers for discussion and action (Figure 1).

## Methods

**Study design:** we conducted a descriptive cross-sectional study using the CDC guidelines for evaluating public health surveillance systems [4].

**Study setting:** Makonde is one of the seven districts in Mashonaland West Province. It lies 118 kilometres North-West of Harare, along the Harare-Chirundu Road. Makonde District serves a total population of 308,739 (*DHIS population projections, 2020*). The economic activities in the district are mining in Mhangura, commercial farming, and subsistence farming in Doma resettlements. Makonde District has a total of 38 health facilities including a provincial referral hospital. The health facilities are comprised of seven government-owned, 28 rural council owned, and 3 urban council-owned health facilities. On average, the health facilities in rural areas are 10 to 25 kilometres apart. The 38 health facilities in the district participate in the WDSS. WDSS is supported by the central government and local authorities in the district namely Chinhoyi Town Council, Mhangura Municipality, and Makonde Rural Council.

**Study population:** the study population were the 44 nurses, and 4 health information officers working in the hospitals and clinics in Makonde District who participated in the WDSS. We reviewed 13 T12 forms, and 335 T3 forms from the 1^st^ of January 2019 to the 31^st^ of December 2020.

**Key informants:** we interviewed the Provincial Epidemiology Disease Control Officer (PEDCO), the Provincial Nursing Officer (PNO), and the Provincial Health Information Officer (PHIO) as key informants. We also interviewed the District Medical Officer (DMO) and the District Nursing Officer (DNO), the Health Information Officers, and the Sister in Charge Community (SICC).

**Sample size:** we calculated the sample size using the Dobson formula. We assumed that 12% of the health workers had good knowledge based on a similar study by Mairosi N *et al*. (2017) in Centenary District, Zimbabwe [5]. We used a confidence interval (CI) of 90%, a delta of 10% and a non-response rate of 10%, a minimum sample size of 46 health workers was calculated. This sample was 25.2% of the total eligible health workers in the study population.

**Sampling:** we selected the ten clinics and three hospitals using simple random sampling by using the RANDBETWEEN Function of the Microsoft Excel program. We selected the health workers using systematic random selection using the line list as a sampling frame. Each health worker was assigned a number, the numbers were then called out and every fourth health worker was selected. The 8 key informants were purposively sampled.

**Data collection:** the questionnaire was pretested at a local clinic school in Zvimba. We conducted interviewer-administered questionnaires to collect data from the health workers. The questionnaire was used to assess the knowledge levels of the health workers on the WDSS, its objectives, and operation; to assess the system attributes and the challenges faced in the operation of the system. A checklist was used to assess the resources required to operate the WDSS. All T12 and T3 forms and charts of the period were reviewed as part of data collection. We conducted interviews with key informants at the school and district education office.

### Definition and measurement of variables based on CDC guidelines for evaluation of surveillance systems

**Data quality and completeness:** the completeness and validity of the data recorded in the public health monitoring system are reflected in data quality [4]. We checked the T3 notification forms for data completeness by making sure that all 37 fields were filled out completely and correctly. Good data quality was defined as having completed all 37 sections, whereas poor data quality was defined as having any of the 37 sections incomplete.

**Flexibility:** a public health surveillance system's flexibility is described as its capacity to adjust to changing information needs or operational conditions with little additional staff, time, or finances [4]. The T12 and T3 notification form's flexibility was evaluated by looking at its capacity to incorporate new information or new notifiable diseases when they were discovered.

**Acceptability:** acceptability is described as people and organizations' willingness to engage in a public health surveillance system [4]. We determined acceptance by asking healthcare workers if they would be willing to participate in the WDSS. We checked for acceptability objectively by looking at the completeness of T12 and T3 notification forms and the timeliness with which T3 forms were reported. We also used a minute book to check on healthcare employees' participation at WDSS meetings.

**Simplicity:** the public health surveillance system's structure and ease of operation are both referred to as simplicity [4]. Simplicity was measured objectively by asking and seeing how easy it was to use the system and fill out the T3 forms. When all of the information needed is accessible, the T3 notification forms should take no more than ten minutes to complete, according to the IDSR training standards used to train healthcare personnel on the WDSS.

**Timeliness:** the speed with which steps in a public health surveillance system are completed is referred to as timeliness [4]. To determine timeliness, we compared the dates on which T1 notification forms were completed and sent to the district to the date on which a notifiable disease was diagnosed. The number of notifiable diseases reported to district offices within 24 hours after diagnosis or reporting was used to determine timeliness.

**Stability:** a public health monitoring system's stability refers to its dependability and availability [4]. The availability of resources, training, and system performance were all used to assess stability. We looked at health-care employees who had received IDSR training that included the WDSS component.

**Usefulness:** a public health monitoring system is useful, according to the new CDC guidelines, if it helps to prevent and reduce adverse health-related events [4]. The participants' assessments of the usefulness of the WDSS, public actions or decisions that were carried out or made based on the findings from the WDSS data were all asked about. Based on WDSS data, we objectively looked for proof of meeting minutes and public acts done.

**Healthcare worker knowledge:** a rating scale was used to assess health worker knowledge, where a rating of poor, fair and good was used [4]. We measured health worker knowledge by the use of eight questions on expected WDSS knowledge. Assuming that each correct response carries the same weight, the responses were graded as poor for 0-3 correct responses, fair 4-6 correct responses, and good for 7-8 correct responses.

**Data analysis:** the data was cleaned and analysed using Epi Info 7.2.4.0^TM^ (CDC, 2020) statistical software. Descriptive statistics were used to describe the study population. The statistical package was used to generate frequencies, means, and proportions. Health worker knowledge of the weekly disease surveillance system was assessed by asking five key questions. Out of the five questions on knowledge, a 3-point Likert scale where the participants who got less than 3 points were rated as poor, those who got 3-4 points were fair and those who got greater than 4 points were good.

**Ethical considerations:** we did not collect any of the study participants' personal information each questionnaire was given a unique number to identify it. We provided the participants with information regarding the study description and participants' rights in the study. We explained the purpose of the study, procedures involved, possible discomforts, and risks. We also explained the potential benefits and the participants' right to withdraw from the study before and during the study and written informed consent was obtained. Ethical approval for the study was obtained from the Mashonaland West Provincial Medical Directorate Institutional Review Board. Permission to proceed was obtained from the Provincial Medical Director (PMD) Mashonaland West, Health Studies Office, and the District Medical Officer.

**Availability of data and material:** the data that support the findings of this study are available from the Ministry of Health and Child Care (Zimbabwe), but restrictions apply to the availability of these data. Data are however available from the authors upon reasonable requests and with permission of the Ministry of Health and Child Care (Zimbabwe).

**Funding:** this study was funded by Mashonaland West Provincial Medical Directorate and Health Studies Office.

## Results

**Demographics:** we interviewed a total of 46 health workers comprising 10 males and 36 females. Of these, 36 (78%) were Registered General Nurses 6 (12%) State Certified Maternity Nurses, and 4 (10%) health information clerks. We noted the median length of experience was 4.0 years (Q_1_=2, Q_3_=8.0) and the median length of working in Makonde District was 2.0 years (Q_1_=1; Q_3_=5) (Table 1). Knowledge. We found that 33 (73%) health workers could name the definition of the system, 28 (60%) could name all the diseases monitored in WDSS, and 40 (88%) knew that the deadline for submission was Monday. However, we found that only 22 (48%) health workers knew at least one of the objectives of the system and only 22 (48%) health workers could name the T series of forms filled in WDSS.

**Table 1:** demographic characteristics of respondents in Makonde District, Zimbabwe, 2020

Variable	Category	Frequency n (%)
Sex	Male	10 (22)
	Female	36 (78)
Health workers	Registered general nurse	36 (78)
	State certified maternity nurse	6 (13)
	Health information clerk	4 (9)
Median years in service	4 (Q1=2; Q3=8)	
Median years working in Makonde District	2 (Q1=1; Q3=5)	

The health cadres involved in the WDSS are nurses and health information officers

**Completeness:** we assessed 335, T3 forms from all the 13 health facilities for data completeness. We found 201 forms out of 335 (60%) had entries that had 100% of the data filled out. Only 4 out of 13 facilities had T1 forms and the rest were using improvised forms.

**Usefulness:** we found 40 (87%) expressed that the surveillance system was useful. However, we found that only 22 (46%) of the respondents mentioned that the data gathered was analysed. We found that only 16 (34%) respondents said that information gathered was used in meetings (Table 2). Only 5 out of 41 respondents had received feedback from the district on the data surveillance system in the past 2 months. We found that data were compiled for transmission purposes, conducting awareness campaigns, and identification and investigation of outbreaks according to those who said it was useful. However, there was no documented evidence in all the centres visited to show that data collected was used at the local level.

**Table 2:** usefulness of the WDSS in Makonde District, Zimbabwe 2020

Variable	Category	Frequency n (%)
Is the Weekly Disease Surveillance system useful	Yes	40 (88)
	No	6 (12)
Meetings to discuss data collected	Yes	16 (34)
	No	30 (66)
Analyse information	Yes	22 (46)
	No	24 (54)
Received feedback from the district in the last 2 months	Yes	5 (13)
	No	41 (87)

**System attributes**

***Acceptability:*** at the clinic level, all nurses had the responsibility of completing the return forms and at the district level, the health information officer had that responsibility. We found that 34 (75%) respondents said completing WDSS forms as part of their job description and 12 (24.1%) said it was the responsibility of the sister in charge. This alone and the completeness of the submitted forms, which was good, was an indication that the system was acceptable to the health workers.

***Stability:*** hand postal services, telephones, cell phones, and radios were the means of communication used in 10 (77%) of the centres. However, 90% of rural clinics did not have landline telephones, and had 80% non-working radios. We found only 2 out of 46 (3%) health workers were trained in disease surveillance. The last district training on surveillance had been conducted more than 5 years prior and all new staff was yet to be trained. We found that 26 (57%) health workers reported stock-outs of the reporting forms.

***Simplicity:*** we noted that 39 (85%) health workers said they had filled in a T1 form and all said they found it easy to fill. All those who had filled in the form said they found it not to be time-consuming at all. When asked to fill in the forms we noted that on average health workers took about 5 minutes to complete one form. Based on the high proportions of workers who had filled in the form with ease, the system was deemed to be simple.

***Flexibility:*** we found that 28 (60%) health workers reported that the WDSS was flexible. Healthcare workers noted that the system easily accommodated reporting on the COVID-19 pandemic. Data on COVID-19 screening, and testing was being transmitted every week.

***Timeliness:*** we found that 31 (65%) of respondents reported that they always send their reports on time every Monday. We however found that higher levels reported that only nine sites out of the thirteen sites (69%) send data on time. This means that the system was not timely at the local level.

**Figure 2:**
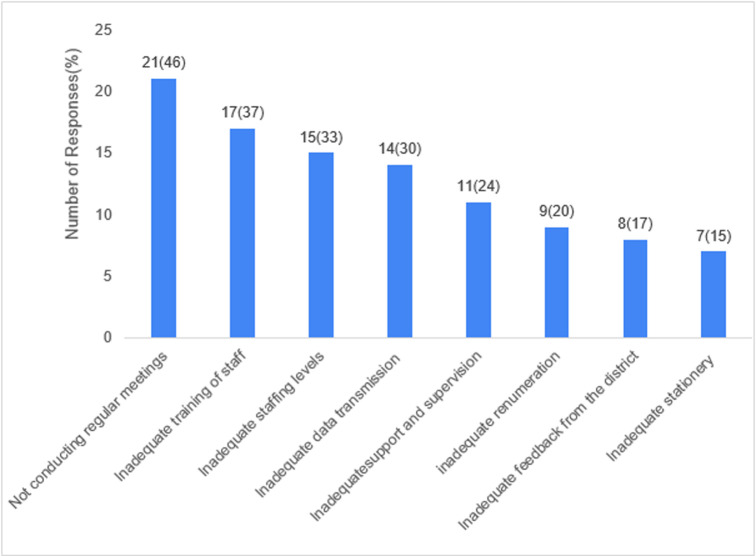
reasons for the non-detection of outbreaks

**Non-detection of outbreaks:** the reasons cited by the respondents as causes of missing outbreaks were not conducting regular meetings (46%), inadequate training of staff (37%), inadequate staffing levels (33%), inadequate data transmission (30%), inadequate support, and supervision (24%), inadequate remuneration (20%), inadequate feedback from the district (17%) and inadequate reporting tools (15%) (Figure 2).

**Key informants:** interviews with District and Provincial executive members as key informants noted that training on disease surveillance had last been done in 2010 in the province and most of the new staff were yet to be trained. The executive members noted that there was a staff shortage in the district with 25% of the health posts were vacant. We found that the district had managed to conduct support and supervision regularly every quarter however the district did not manage to reach every clinic each quarter.

## Discussion

The introduction of novel infectious diseases, as well as the recurrence of existing ones, has made disease surveillance essential [6,7]. It is also important that program managers utilise the data they gather to respond to outbreaks timeously and implement mitigatory measures to curb the spread of the disease. We evaluated the weekly disease surveillance system in Makonde District on the reasons for missing outbreaks, knowledge of health workers regarding the WDSS, the WDSS system attributes, and the usefulness of the WDSS. The surveillance system was simple, flexible, acceptable, but unstable, untimely, and not useful. There was poor knowledge on the surveillance system, health workers were not trained on disease surveillance, and quality of data was poor.

The health workers' poor knowledge of the system's objectives could be attributed to inadequate training on disease surveillance. These findings are similar to Wang *et al*. in China who stated that there was under-reporting of the notifiable infectious diseases due to poor knowledge in the surveillance system reporting [7]. Studies in Nigeria [8] and Zimbabwe [9-11] noted that the health workers' knowledge of surveillance systems was fair to poor, hence the need for training in surveillance systems. The usefulness of a surveillance system denotes how it is used and the decisions that are taken using the information obtained from it. We found it to be not useful in informing decisions. These findings could be attributed to poor staff attitude [5], and inadequate reporting tools available [12]. These findings were similar to a study by Muringazuva *et al*. [10] in Kadoma who found that the city did not use the data gathered in meetings and no data was analysed.

We noted the district was not utilising the data gathered in the WDSS. Health workers in the district were merely gathering data and reporting it to the next level with very little analysis on-site through meetings. This finding is similar to multiple studies on surveillance systems in Zimbabwe where inadequate healthcare worker training on surveillance [8], limited coordination of surveillance activities [9], insufficient reporting resources [10], and competing work priorities were possible reasons for inefficient surveillance systems [11]. These weaknesses of the surveillance system greatly hamper efforts to detect outbreaks timeously.

**System attributes:** data quality reflects the completeness and validity of the data recorded in the WDSS [13]. We found that the poor data quality in Makonde District could be attributed to the inadequately filled reporting forms. Similarly, effective disease surveillance, notification, and reporting have been a serious challenge in developing countries [14] due to the lack of trained personnel and inadequate resources. Njuguna *et al*. in Sierra Leone, found that data quality issues such as data incompleteness affected the validity of the mortality estimates [15].

Flexibility is the ability of the WDSS to adapt to changing information needs or operating conditions with little additional time, personnel, or allocated funds [16]. In Makonde, we noted the system was found to be stable. The system had seamlessly incorporated new emerging diseases such as SARS CoV-2 further highlighting its flexibility. Similarly, Tsitsi *et al*. noted that health workers in Beitbridge found the system flexible [16].

Acceptability is the willingness of persons and organizations to participate in the WDSS [17]. The system was acceptable in the district although some health workers did not feel it was their duty to report data. The reasons cited were inadequate training and they felt it was the duty of supervisors. These findings are similar to Constantine *et al*. in Chimanimani who found that health workers reported that they feared using the AEFI surveillance system due to concern over investigations that followed [18]. Stability refers to the ability of the WDSS to collect, manage, and provide data without failure and to be operational when needed [4]. In this study, the instability of the surveillance system could be attributed to the stock out of reporting forms and that the system was dependent on paper records. Similarly, Makoni *et al*. in Gokwe found that notification forms were only present in half of the district's clinics [9]. This contrasted with the findings of Muringazuva *et al*. [10] in Kadoma who found the surveillance system stable. Health workers reported that notification forms, working telephones, and pens were never out of stock in their city.

Timeliness reflects the speed between steps in the WDSS [19]. For efficient data flow, good communication systems need to be in place. During the study, we noted health workers interviewed reported that the system was efficient in terms of timeliness. However, interviews with the district medical officer and provincial health information officer showed that the system was in contrast not timely. In a study in Chipinge, the use of cell phones to transmit data was noted to significantly increase the timeliness of surveillance systems [2]. However, despite using cell phones to transmit data, poor network connectivity in the district greatly hampered data transmission.

Simplicity refers to both structure and ease of operation of the WDSS [8]. We found the system in the district simple. These findings are similar to studies by Benson, in South Africa [20] and Mairosi *et al*. [5] who noted that stakeholders who were younger than 35 years and those who were trained in WDSS found the system to be simple.

**Limitations:** while these are useful findings, they are limited by only including Makonde District. This does not ensure these interventions would be as effective in other districts. Similar studies, in the province, would help to further clarify the methods.

## Conclusion

The weekly disease surveillance system in Makonde District was reported to be simple, flexible, acceptable, sensitive, and representative. There was poor knowledge of the surveillance system's objectives among the respondents. We noted that health workers in the district were not trained on disease surveillance. The quality of the data was poor, and the district did not consider the system as useful. We found the system was not stable, untimely, and not useful. We recommend that should ensure that all health workers in the district are trained in IDSR and have adequate reporting tools. To stimulate health worker interest and improve on timeliness the district should provide regular feedback and be trained to validate the data to ensure there are no missing details.

### What is known about this topic

- The weekly disease surveillance system is a tool used to provide an early warning of potential public health threats in Zimbabwe;
- The system is a passive surveillance system used in detecting outbreaks of infectious disease.

### What this study adds

- Healthcare workers in Makonde District did not consider the weekly disease surveillance system to be useful in their day-to-day operations and they did not use it to detect outbreaks.
